# Costimulatory Molecules on Immunogenic Versus Tolerogenic Human Dendritic Cells

**DOI:** 10.3389/fimmu.2013.00082

**Published:** 2013-04-03

**Authors:** Mario Hubo, Bettina Trinschek, Fanny Kryczanowsky, Andrea Tuettenberg, Kerstin Steinbrink, Helmut Jonuleit

**Affiliations:** ^1^Department of Dermatology, University Medical Center of the Johannes Gutenberg-University MainzMainz, Germany

**Keywords:** dendritic cells, tolerance, immunity, IL-10, regulatory T cell, costimulation, inhibitory molecules

## Abstract

Dendritic cells (DC) are sentinels of immunity, essential for homeostasis of T cell-dependent immune responses. Both functions of DC, initiation of antigen-specific T cell immunity and maintenance of tissue-specific tolerance originate from distinct stages of differentiation, immunogenic versus tolerogenic. Dependent on local micro milieu and inflammatory stimuli, tissue resident immature DC with functional plasticity differentiate into tolerogenic or immunogenic DC with stable phenotypes. They efficiently link innate and adaptive immunity and are ideally positioned to modify T cell-mediated immune responses. Since the T cell stimulatory properties of DC are significantly influenced by their expression of signal II ligands, it is critical to understand the impact of distinct costimulatory pathways on DC function. This review gives an overview of functional different human DC subsets with unique profiles of costimulatory molecules and outlines how different costimulatory pathways together with the immunosuppressive cytokine IL-10 bias immunogenic versus tolerogenic DC functions. Furthermore, we exemplarily describe protocols for the generation of two well-defined monocyte-derived DC subsets for their clinical use, immunogenic versus tolerogenic.

## Introduction

### Dendritic cells – sentinels of immunity

Ralph Steinman started as a postdoc in the laboratory of Zanvil Cohn and James Hirsch at the Rockefeller University in the 1970s. The focus of his research was the identification and functional characterization of dendritic cells (DC) granted in 2011 with the Nobel Prize for medicine. Steinman identified this novel cell type in murine spleens and thereby opened a complete new field in immunology. The link between innate and adaptive immunity was revealed, concomitantly the origin of antigen-specific T cell-mediated immune responses (Steinman, 2012).

The family of DC is divided into two major subtypes with distinct functions: plasmacytoid and conventional DC. Plasmacytoid DC express receptors for recognition of viral antigens and produce high amounts of type I interferons after activation. Thus, the main function of this DC subtype is the initiation of anti-viral responses. Conventional DC are further divided into numerous subtypes residing in specific tissues in an immature state. They express a broad range of receptors for recognition of bacterial and viral components (Wu and Liu, 2007).

**Figure 1:**
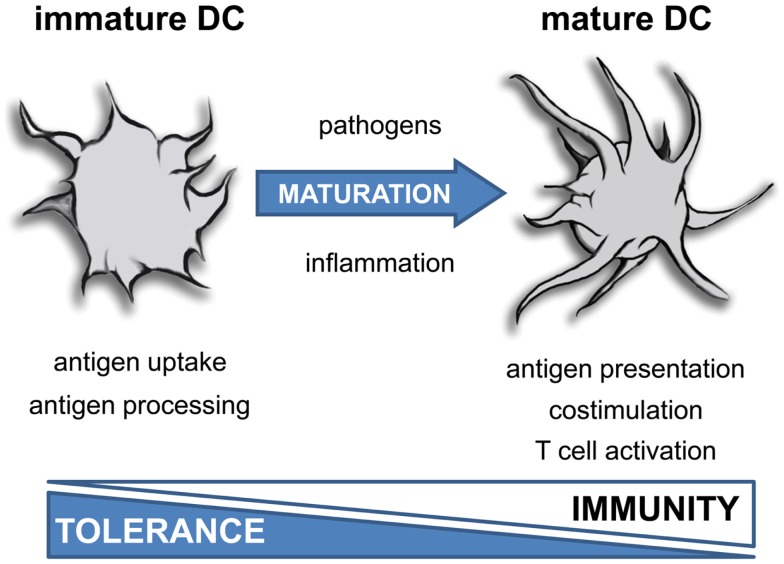
**Function of dendritic cells depends on maturation**. Inflammatory mediators induce terminal differentiation of immature DC into fully matured immunogenic DC. This process is associated with a dramatic change in morphology, a reduced uptake of antigens and impaired antigen processing activity. Furthermore, mature DC exhibit a strong costimulatory and T cell activating capacity.

Dendritic cells turned out to be uniquely equipped for activation of naïve T cells and therefore are referred to as “professional” antigen-presenting cells. They are located in nearly all peripheral tissues. Here, immature DC differentiate from blood-derived progenitors under the influence of tissue-specific factors. Tissue residing DC form a close network, optimally positioned to sense invading pathogens. They excessively capture antigens by phagocytosis, macropinocytosis, or receptor-mediated endocytosis and further process these antigens into peptides. The peptides are loaded onto major histocompatibility complex (MHC) molecules and finally presented on DC surface. Due to their strong migratory capacity, antigen taken up by immature DC in the periphery is efficiently transported to T cell areas of local lymph nodes (Banchereau and Steinman, 1998) (Figure 1). Here, antigens are presented to T cells, which results in tolerance in absence of inflammation or immunity under inflammatory conditions. Therefore, the constant migration of immature DC to lymph nodes and the presentation of self-antigens are crucial parts of maintenance of peripheral tolerance. Under this aspect, it is not surprising that the vast majority of DC found in lymphoid organs under steady state conditions exhibit an immature phenotype (Wilson et al., 2003). These immature DC constitute of migratory immature DC from the periphery and tissue resident lymphoid DC (Shortman and Naik, 2007).

Recent reports showed that DC not only determine the type of T cell immunity, but also patterns of homing receptors expressed on T cells and thus their migratory behavior (Dudda and Martin, 2004; Sigmundsdottir and Butcher, 2008; Schwarz et al., 2011; Naik et al., 2012). Blood-derived DC mostly express both gut and skin homing markers and, thus, are able to migrate to both organs. These DC induce T cells with multi-homing properties. After immigration into particular tissues, DC within gut or skin do not further exhibit this ability and induce rather tissue-specific T cells. These functional changes of DC are a result of tissue-specific maturation processes (Johansson-Lindbom et al., 2003).

### Dendritic cells as potent inducers of immunity and tolerance

Dendritic cell function strictly depends on their current activation state. Under steady state and dependent on their localization, DC display an immature phenotype that correlates with low expression of costimulatory molecules and weak T cell stimulating properties (Banchereau and Steinman, 1998). Furthermore, functional properties of DC subsets are adapted to tissue functions. Particular tissues benefit from the unique capability of DC to either induce antigen-specific responses or tolerance. DC located in the mucosa of lung or gut are confronted with a continuous influx of foreign antigens. Mediated by tolerogenic mediators like IL-10 and TGF-β, the local micro milieu strongly prevents DC activation to avoid pathologic inflammation and DC in these environments rather promote tolerance than immunity (Akbari et al., 2001; Weiner, 2001). In contrast, lymphnodes and blood are protected against uncontrolled influx of antigens and the local environment lacks tolerogenic mediators. Immature DC located in lymph nodes and blood likewise maintain peripheral tolerance, but as a consequence of a different local milieu, these DC need less stimulation for maturation into immunostimulatory DC (Iwasaki and Kelsall, 1999).

Pathogens exhibit a broad range of molecular patterns that are recognized by specific receptors such as Toll-like receptors (TLR) expressed by DC. Direct recognition of invading pathogens activates immature DC and induces their differentiation. In addition to these pathogen-triggered signals, local inflammation influences the differentiation process of DC (Medzhitov, 2001).

**Figure 2:**
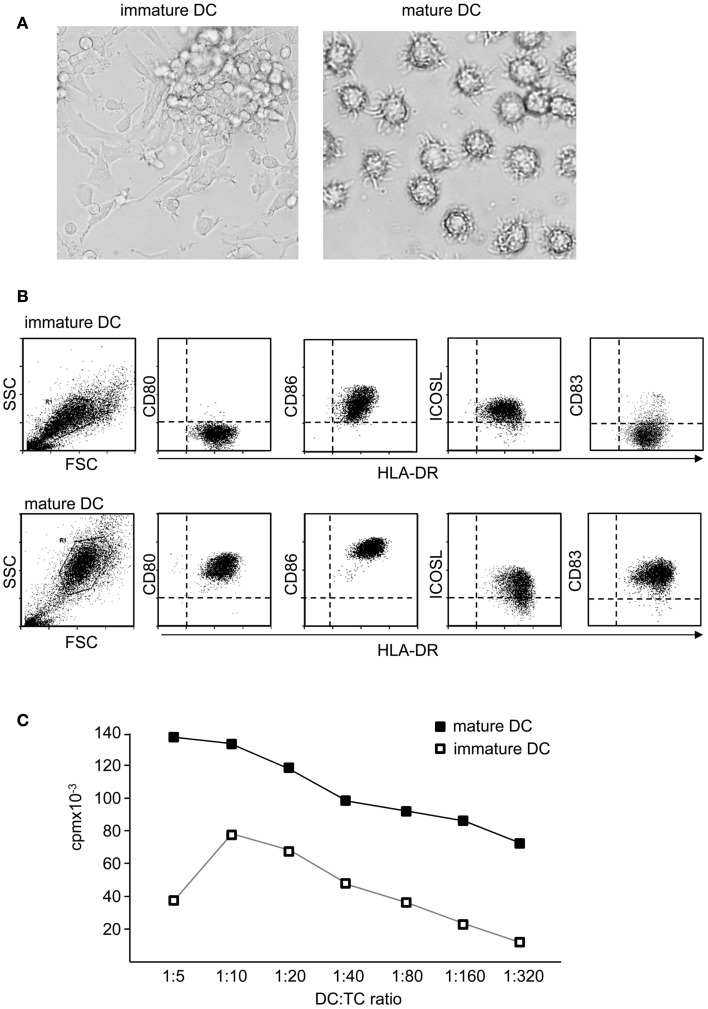
**Mature dendritic cells are potent activators of naïve T cells**. **(A)** Functional properties of DC depend on their maturation state. In contrast to immature DC, terminally differentiated DC show a typical morphology with strong cellular extensions and **(B)** induce specific maturation markers like CD83 and costimulatory molecules like CD80 or CD86, whereas ICOSL is rather down regulated or unaltered. Also antigen presentation is enhanced, displayed by higher levels of MHC molecules. **(C)** In coculture with alloreactive T cells, immature DC induce only comparable weak T cell proliferation, whereas mature DC are potent activators of T cells.

As a result of maturation, DC undergo a dramatic change in their morphology and develop cellular extensions that enlarge cellular surface and improve the interaction with T cells (Figure 2A). DC also downregulate IL-10-receptor (IL-10R) expression rendering them insensitive to the immunosuppressive function of this cytokine (Steinbrink et al., 1999; Thurner et al., 1999). But the major events in DC maturation are probably the upregulation of MHC and costimulatory molecules on their surface (Figure 2B). The maturation process also drastically enhances their migratory capacity. Through upregulation of homing receptors like CCR7, migration to lymph nodes is accelerated. Those migratory DC follow gradients of chemokines such as CCL19 and CCL21 and enter T cell areas of secondary lymphoid organs (Dieu et al., 1998; Sallusto and Lanzavecchia, 2000). Importantly, activated DC cease any further uptake and procession of antigens. This ensures that antigens which are transported and presented by activated DC reflect the current situation at the site of inflammation. Assimilation of self-antigens on the way to lymph nodes and subsequent activation of self-reactive T cells are thereby prevented. Altogether, these events render mature DC potent inducers of T cell proliferation (Figure 2C) and T cell differentiation.

Activation of naïve T cells requires several distinct signals delivered by DC: signal I is mediated by MHC in complex with a peptide processed from captured antigens and is received by a specific T cell receptor. For entire T cell activation a costimulatory signal (signal II) is mandatory, as a T cell receptor signal in absence of costimulation renders respective T cells anergic (Corthay, 2006). In addition, a third signal in form of soluble factors such as IL-12, IL-15, IL-6, or TNF-α is also important for functional activation of naïve T cells. An integration of all signals designs the T cell differentiation process: inflammatory versus tolerogenic (Curtsinger et al., 1999). In strong contrast to naïve T cells, reactivation of effector or memory T cells is rather signal II independent, ensuring rapid execution of effector function at sites of inflammation independent of accessory cells (Byrne et al., 1988; Croft et al., 1994).

## Impact of Signal II on DC Function

**Figure 3:**
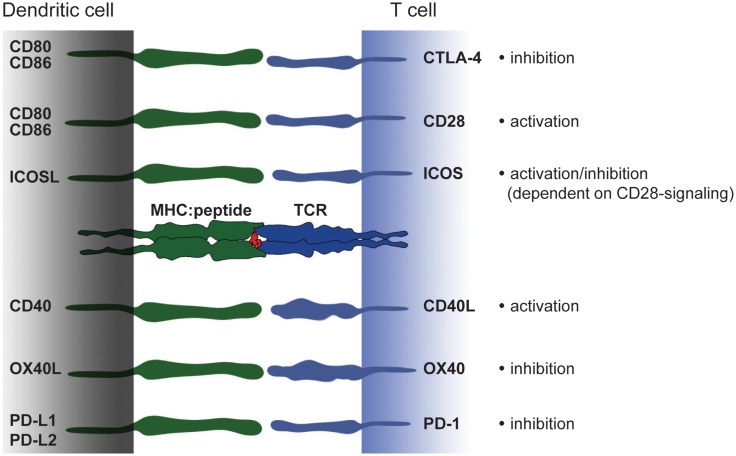
**Costimulatory molecules and their ligands – a brief overview**. Modulation of T cell activation is mediated by an interplay of different costimulatory molecules expressed on DC that have either immunogenic or tolerogenic function. The picture shows an overview of members from the B7 and TNF-receptor family expressed on DC and their binding partners on T cells. In the last decade a number of new costimulatory molecules have been identified. However, in the context of monocyte-derived DC CD80 and CD86 constitute powerful members of the costimulatory family. Strong CD80/CD86-derived signals can overcome e.g., ICOSL-mediated signaling and thereby turning a rather tolerogenic signal into an immunogenic.

Pattern of costimulation hence is a central feature distinguishing tolerogenic and immunogenic DC. But it is not solely absence or presence of costimulation that defines DC function. A complex network of transmembrane receptor/ligand pairs acts together with the T cell receptor and soluble factors to enhance T cell activation (Figure 3). Under these molecules, CD28, ICOS, and CD40L play a prominent role. At the same time, T cells also express inhibitory molecules such as CTLA-4 or PD-1, that down regulate T cell activation. Ultimately, it is the combination of several circumstances including the subtle interplay of signal II that produces an immunogenic or tolerogenic immune response. Here, we give a short overview of costimulatory molecules from the B7 family and TNF-receptor family that either support tolerogenic or immunogenic function.

### Costimulatory molecules of the B7 family

#### CD80/CD86

CD80 (B7.1) and CD86 (B7.2) expression on DC probably constitutes the most important costimulatory pathway in T cell activation (Lenschow et al., 1996). Signaling through binding partner CD28 on T cells confers optimal mRNA stabilization and production of IL-2, a factor that promotes expansion and survival of primary T cells (Linsley et al., 1991). A variety of inflammatory or pathogen-derived mediators quickly up regulate expression of CD80 and CD86, therefore both molecules serve as very early costimulatory signals (Figure 2B). CD28-mediated costimulation also strongly interferes with tolerogenic properties of immature DC. A strong CD28 signal can inhibit differentiation into induced Treg by preventing stabilization of IL-10R on T cells (Tuettenberg et al., 2009). Interestingly, the same costimulatory molecules are also responsible for shutting down T cell activation. This is realized by a simple trick: T cell activation is accompanied by upregulation of CTLA-4 on T cell surface. CTLA-4 binds with higher affinity to CD80/CD86 than CD28 and thereby competes for interaction with both costimulators. CTLA-4-mediated signaling down regulates T cell responses and thus, provides a very simple negative feedback loop carried out by the same ligand (Greene et al., 1996; Carreno et al., 2000).

Both, CD80 and CD86 are commonly used for describing fully matured DC. Often this is conveyed between species, a fact that has to be handled with care. *Human* immature DC constitutively express intermediate amounts of CD86 and lack CD80 (Figure 2B) (Jonuleit et al., 1997). Hence, for characterization of *human* DC maturation, CD80 is considerably more reliable, as it is exclusively induced on mature DC while CD86 is already present on immature DC and further up-regulated upon stimulation. In contrast, in the murine system CD86 is the main activation marker of bone-marrow derived DC, strongly up-regulated after maturation (Inaba et al., 1992, 1994) while CD80 expression is less pronounced on *murine* DC.

#### ICOS-Ligand

ICOS is expressed on CD4^+^ T cells upon T cell receptor-mediated activation (Hutloff et al., 1999) and specifically interacts with ICOS-Ligand (ICOSL; B7-H2) on antigen-presenting cells (Yoshinaga et al., 1999). ICOS regulates general T cell features such as growth, proliferation and survival. In addition, depending on the inflammatory environment, ICOS/ICOSL interaction drives T cell polarization (Kopf et al., 2000).

**Figure 4:**
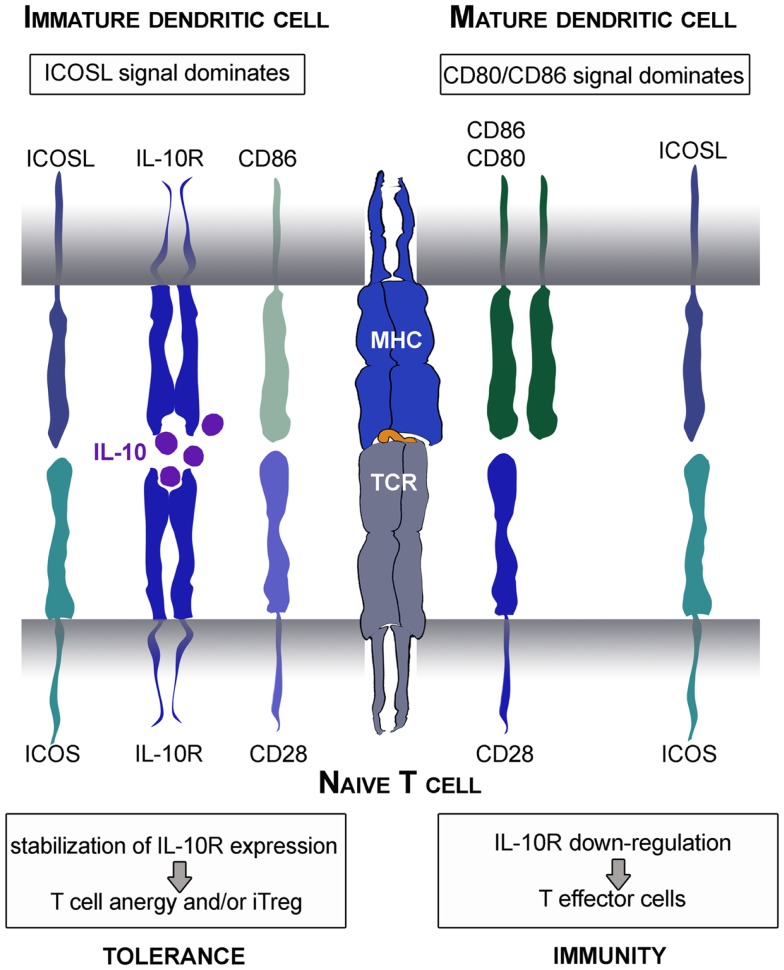
**Tolerogenic or immunogenic function of ICOS/ICOSL interaction depends on CD28 signaling**. Immature DC express low amounts of CD28 ligands and thereby provide strong ICOS signals. This leads to stabilization of IL-10R on the surface of stimulated naïve T cells. Subsequently, the immunomodulatory cytokine IL-10 produced by DC mediates its function resulting in the differentiation into anergic Treg. In contrast, mature DC provide strong CD28 signals that overcome the tolerogenic ICOS function resulting in stabilization of IL-2 mRNA and synthesis and thereafter, the differentiation into inflammatory T effector cells.

Moreover, a central role for ICOS in mediating tolerance has been suggested in mouse and men (Rottman et al., 2001; Herman et al., 2004). In view of this aspect, it is interesting that immature *human* DC express high amounts of ICOSL on their surface (Figure 2B). This is an important fact, as immature DC thereby convey a strong ICOS-signal in context of weak CD28-stimulation which was shown to stabilize IL-10R-expression on stimulated T cells. Under these circumstances, low amounts of IL-10 produced by immature DC act on IL-10-sensitized T cells allowing immunosuppressive functions that prevent differentiation into inflammatory T effector cells (Figure 4). Altered T cell polarization results in low proliferative capacities and production of IL-10 instead of IFN-γ. Finally, these T cells differentiate after repetitive stimulation into induced Treg (Jonuleit et al., 2000b). This process is again driven by the balance of distinct engaging costimulatory signals: the induction of IL-10-producing Treg critically depends on ICOS/ICOSL interaction and is prevented by strong CD28 signaling (Witsch et al., 2002; Tuettenberg et al., 2009). Interestingly, activated human plasmacytoid DC express high levels of ICOSL and rather low CD28 ligands. Also this DC subset promoted differentiation of naïve T cells into IL-10-producing regulatory T cells in an ICOS-dependent fashion (Ito et al., 2007). This again illustrates the plasticity of a DC-derived immune response, as we showed recently that the same population of plasmacytoid DC is also able to elicit T cell proliferation in presence of regulatory T cells (Hubo and Jonuleit, 2012). Therefore it is important to note, that DC function cannot be attributed to the expression of single molecules but has always to be considered in the context of the local milieu.

Several groups reported a central role for ICOS-mediated costimulation in tolerance also in *mice*. Here, interaction of ICOS/ICOSL is required for Treg induction (Busse et al., 2012) or for maintenance of peripheral tolerance (Rottman et al., 2001; Herman et al., 2004). Taken together, ICOS/ICOSL interaction plays an important role in development of adaptive tolerance by DC rendering ICOS an interesting target for immunotherapy.

#### PD-1 ligands

Programed cell death-1 (PD-1) has two ligands, PD-L1 and PD-L2. PD-L1 is constitutively expressed on resting DC as well as on other immune and non-immune cells (Yamazaki et al., 2002). After stimulation of immature DC with pathogen-derived factors like LPS or after CD40-mediated signaling, PD-L1 expression is further enhanced. Compared to PD-L1, PD-L2 expression is restricted to antigen-presenting cells like B cells, macrophages and DC (Zhong et al., 2007). Here, the molecule is up-regulated in response to anti-CD40, GM-CSF, IL-4, IFN-γ, and IL-12 (Loke and Allison, 2003). Interaction of T cells and DC via PD-L/PD-1-axis transfers inhibitory signals into T cells by inhibiting activation of PI3K. Subsequently, production of cytokines like IFN-γ is repressed, cell survival proteins are impaired and apoptosis is induced (Keir et al., 2008).

One mechanism of tolerogenic DC to shut down self-reactive T cells in the periphery is achieved through PD-1 signaling by induction of Treg. Just like ICOS/ICOSL-mediated induction of Treg, also the tolerogenic function of PD-1 underlies similar immune mechanisms: strong costimulation delivered by mature DC via CD28 overcomes PD-1-mediated inhibitory effects (Chemnitz et al., 2004). In summary, PD-1 signaling down regulates immune responses and so participates in peripheral tolerance (Nishimura et al., 2001; Krupnick et al., 2005).

In general, inhibitory effects in the immune system have a high potential to become pathologic, e.g., within a growing tumor. Cancer has generated several mechanisms to efficiently evade immune responses; among others overexpression of inhibitory molecules is critical. PD-1L was found to be expressed in high amounts on a multitude of solid tumors (Hamanishi et al., 2007; Nakanishi et al., 2007) thereby provoking a suppressive microenvironment that was suggested to explain the failure of anti-tumor immunotherapies. Also numerous autoimmune diseases such as type I diabetes, multiple sclerosis, systemic lupus erythematosus, and rheumatoid arthritis are linked with dysregulated PD-1 shown by analysis of single-nucleotide polymorphisms (Prokunina et al., 2002; Ferreiros-Vidal et al., 2004). Thus, targeting this costimulatory pathway might be beneficial to generate new therapies.

### Costimulatory molecules of the TNF-receptor family

#### CD40

During interaction of DC and T cells, further receptor-ligand pairs are up-regulated and new possibilities for T cell modulation develop. These molecules include CD40L (CD154; member of the TNF superfamily) on activated T cells and CD40 expressed by activated DC and other antigen-presenting cells (Grewal and Flavell, 1998).

The CD40/CD40L pathway regulates cellular and humoral immunity and plays an important role in T cell priming and differentiation (MacDonald et al., 2002). Using blocking antibodies and knockout models, CD40/CD40L interaction was shown to be required for protective immunity (Reichmann et al., 2000; Habib et al., 2007). CD40 ligation on DC increases expression of costimulatory, adhesion and MHC molecules and promotes the production of T cell stimulatory cytokines such as IL-12 (Lapteva et al., 2007; Haenssle et al., 2008). Recombinant CD40L therefore is often used to induce DC maturation. However, CD40/CD40L interaction alone is insufficient for induction of the important effector molecule IL-12 in human DC. Additional IFN-γ, produced during DC-T cell crosstalk is required for IL-12 production. Since naïve T cells do not produce IFN-γ, their activation by mature DC does not result in IL-12 production by DC (Snijders et al., 1998). Nevertheless, some reports show that CD40-stimulated DC, despite their mature phenotype, induce T cell anergy (Wiethe et al., 2003) via IL-10 production and stabilization of IL-10R on T cells (Tuettenberg et al., 2010). This is not only true for conventional DC but also for plasmacytoid DC that produce large amounts of IL-10 after CD40L activation, resulting in induction of Treg (Gilliet and Liu, 2002).

In *mice* it was shown that also the level of CD40L expression influences the intensity of DC-T cell interaction and thereby modulates the outcome of an immune response. Low CD40L expression on T cells induces IL-10 production that impairs T cell expansion and antigen reactivity. Such anergized T cells were able to gain capabilities to suppress T cell activation. In contrast, strong interaction mediated by high levels of CD40L rather induced IL-12 production thus promotes immunity (Murugaiyan et al., 2007).

In summary, the T cell response resulting from CD40/CD40L interaction is complex and strictly depends on signal strength and presence of third signals such as IFN-γ that favor T cell-dependent immunity or tolerance.

#### OX40 ligand

OX40 ligand (OX40L) is ubiquitously expressed on a multitude of antigen-presenting cells. In addition, also non-immune cells like endothelia and smooth muscle cells show OX40L expression (Imura et al., 1996; Ohshima et al., 1997; Burgess et al., 2004). OX40L is induced on DC after CD40L stimulation or in response to inflammatory mediators like TNF-α (Ohshima et al., 1997; Migone et al., 2002; Fillatreau and Gray, 2003). OX40L signaling plays a central role in immunity and tolerance, controls T cell survival and homeostasis and at least supports the generation of long-lasting memory T cells. Interestingly, OX40L signaling preferentially promotes Th2 differentiation in the absence of IL-12 and independent of IL-4 (Flynn et al., 1998; Ito et al., 2005). However, together with IL-12, OX40L-mediated T cell stimulation rather induces Th1 polarization (De Smedt et al., 2002).

Besides its potent costimulatory potential regarding T cell activity, OX40/OX40L interactions are also capable to modulate peripheral tolerance in mice and men. Interestingly, Vu et al. reported that Treg stimulation via OX40 represses Foxp3 expression and thereby leads to reduced Treg function. In addition, it was shown that strong OX40-mediated signaling prevents TGF-β-promoted Foxp3 induction and further differentiation into induced Treg (Ito et al., 2006; Vu et al., 2007). Therefore, it is not remarkable that OX40/OX40L interference became a therapeutic target in immune-mediated diseases and cancer. Several studies have proved the protective effect of blocking the OX40/OX40L interaction in models of inflammatory disease like asthma, arteriosclerosis as well as in autoimmune diseases such as experimental autoimmune encephalomyelitis, diabetes, colitis, and collagen-induced arthritis (Croft, 2009; Kaur and Brightling, 2012).

In conclusion, expression of costimulatory molecules on DC is essentially involved in controlling T cell differentiation and the resulting immune response: immunity versus tolerance. No single costimulatory molecule is tolerogenic or immunogenic. It is rather the integration of several costimulatory molecules, interaction with soluble co-factors and the differentiation state of interacting T cells that dictates the immune response.

## IL-10 – Mediator of Tolerance

**Figure 5:**
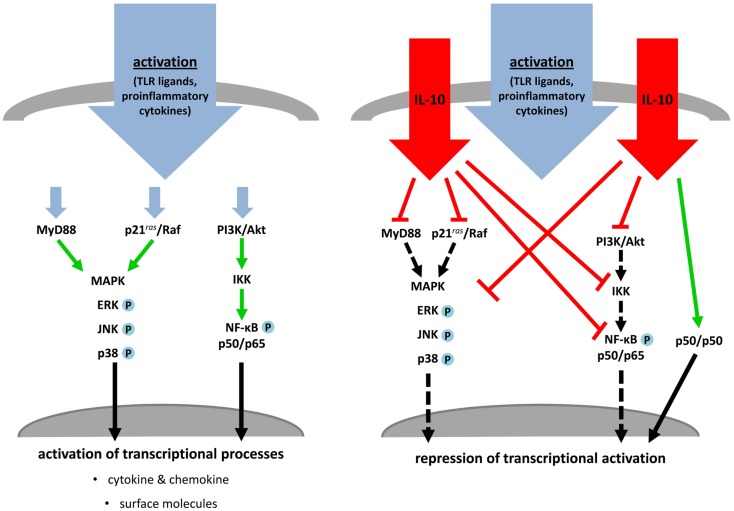
**IL-10 treatment blocks activation and maturation of dendritic cells and monocytes**. TLR ligands or proinflammatory cytokines stimulate MAPK cascades, MyD88- and PI3K/NF-κB-dependent signaling, which induce transcriptional processes resulting in activation and maturation of stimulated APC (left). Simultaneous treatment of these activated immune cells with IL-10 inhibits key molecules of the named pathways. MAPK- and MyD88-dependent signaling and NF-κB activation are directly or indirectly prevented by IL-10. This leads to an abrogation of transcriptional and post-translational processes or induction of inhibitory transcriptional mechanisms (right).

One key soluble molecule that critically contributes to peripheral tolerance by modulation of DC costimulation is the immunosuppressive cytokine IL-10. IL-10/IL-10R-signaling is mediated by activation of the Jak/STAT pathway through tyrosine phosphorylation of Tyk2/Jak1, resulting in downstream activation and homodimerization of STAT3. These dimers are crucial transcription factors for regulation of anti-inflammatory genes (Finbloom and Winestock, 1995; Ding et al., 2003). In the context of DC maturation by several stimuli the most important pathways affected by IL-10 are PI3K/Akt/NF-κB-, MyD88/MAPK-, and Ras/Raf/MAPK-regulated signal transduction cascades (Figure 5). Early inhibition of the src kinase p56lyn and suppression of later events of this PTK-mediated activation (Ras, ERK, p38) are demonstrated for human monocytes and DC (Geng et al., 1994; Niiro et al., 1998; Sato et al., 1999). In addition, TLR-induced expression of the MyD88-dependent adaptor molecules IRAK4 and TRAF6 are post-transcriptional regulated by IL-10 and is associated with decreased phosphorylation of the MAPK p38 and JNK (Chang et al., 2009; Knödler et al., 2009). LPS- or TNF-α-induced activation of NF-κB is also blocked by IL-10 in versatile manner. Inhibition of the PI3K pathway by IL-10 is mediated by abolishment of Akt phosphorylation, abrogation of IκB degradation, blocking of inhibitor of κB kinase (IKK) activity and prevention of NF-κB translocation and DNA binding activity as demonstrated by several groups. In addition to IL-10-induced inhibition of NF-kB-subunit p65 translocation (Shames et al., 1998; Schottelius et al., 1999; Bhattacharyya et al., 2004), IL-10 initiates formation of p50 homodimers which are capable to inhibit transcriptional activity (Driessler et al., 2004).

**Figure 6:**
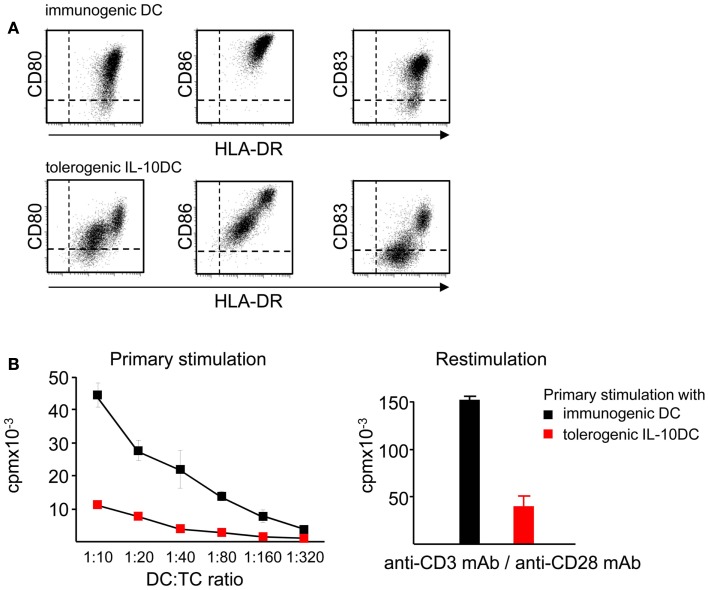
**IL-10 affects maturation and induces tolerogenic DC**. **(A)** Presence of IL-10 during the maturation process of immature DC impairs the upregulation of costimulatory molecules like CD80 and CD86 resulting in the tolerogenic phenotype of IL-10DC. **(B)** Tolerogenic IL-10DC display a reduced T cell stimulatory capacity compared to immunogenic DC (left) and induce T cell anergy, displayed by reduced T cell proliferation after polyclonal restimulation (right).

Maturation of DC in presence of IL-10 induces two distinct subsets of tolerogenic DC (IL-10DC) characterized as CD83^high^CD80/CD86^high^HLA-DR^high^and as CD83^low^CD80/CD86^low^HLA-DR^low^ (Figure 6A). Expression of surface molecules associated with DC maturation like CD83, the lymph node homing receptor CCR7 and MHC class-II molecules is reduced on IL-10DC. In contrast, expression of inhibitory receptors such as immunoregulatory receptors like ILT2, ILT3, and ILT4 are increased on IL-10DC, which has been suggested to be involved in tolerance induction (Velten et al., 2004; Boks et al., 2010, 2012; Torres-Aguilar et al., 2010). The profile of cytokine release of IL-10DC differs according to protocols used for DC generation. Nevertheless, the amount of proinflammatory cytokines like IL-6 and TNF-α is similar as compared to classically matured DC. In contrast, IL-10 production is dramatically increased suggesting another possible mechanism resulting in tolerance induction (Velten et al., 2004; Boks et al., 2010, 2012; Gregori et al., 2010; Torres-Aguilar et al., 2010; Xiuling et al., 2010). Several studies showed that *in vitro* generated tolerogenic IL-10DC have an even higher capacity to induce iTreg than tissue resident immature DC (Steinbrink et al., 1997; Torres-Aguilar et al., 2010; Boks et al., 2012). Thus, a reduced expression of immunostimulatory molecules combined with a higher expression of inhibitory costimulators and the production of IL-10 may facilitate tolerogenic functions of IL-10DC.

The tolerogenic function of IL-10DC was demonstrated by induction of antigen-specific anergy in CD4^+^ and CD8^+^T cells (Kubsch et al., 2003). This anergic state is characterized by low T cell proliferation and decreased IL-2 and IFN-γ secretion (Steinbrink et al., 1999; Steinbrink, 2002) (Figure 6B). In addition, IL-10DC-primed anergic CD4^+^ and CD8^+^T cells exhibit suppressive capacity and are able to inhibit activated Th1, Th2, and Tc1 T cell responses (Steinbrink et al., 1997, 1999; Xiuling et al., 2010). Compared to tolerogenic tissue resident DC, IL-10DC are terminally differentiated and exhibit a stable phenotype. Hence, they are insensitive to conversion into immunostimulatory DC mediated by inflammatory factors. These properties provide the opportunity to use this tolerogenic DC subpopulation for induction of tolerance in the context of autoimmunity. The inflammatory environment exhibits the capacity to induce maturation of immature DC leading to enhanced immune responses, while IL-10DC do not further respond to these signals (Steinbrink et al., 1999; Thurner et al., 1999).

Gregori et al. (2010) confirmed the *in vivo* existence of IL-10DC in peripheral blood of humans. This is in line with patients suffering from hyper-IgE syndrome due to defective STAT3-signaling, a central component of the IL-10-pathway. DC from these patients are insensitive to IL-10, and therefore, show reduced upregulation of inhibitory molecules (PD-L2, ILT3, and ILT4) in response to IL-10 correlating with an impaired capacity to induce Treg (Saito et al., 2011). Thereby a crucial role of tolerogenic IL-10DC for control of immune responses *in vivo* can be suggested. Modulation of immature DC with IL-10 might be a potential approach to develop novel DC-based vaccination strategies to control and limit harmful T cell responses by antigen-specific induction of potent Treg.

## Protocols for Generation of Tolerogenic and Immunogenic Dendritic Cells

Immunogenic DC are currently applied in various clinical studies to evoke anti-tumor immunity in cancer patients. Although immunological responses are often reported, these are mainly transient demonstrating that DC-based vaccines face several barriers limiting their effectiveness in clinical trials (Engell-Noerregaard et al., 2009). Also the use of tolerogenic DC has an important impact in clinical trials to suppress autoaggressive T cell activity in autoimmune diseases or unwanted responses to allergens. Whereas vaccination with immunogenic DC has been tested over the past decade (Schuler-Thurner et al., 2000; Jonuleit et al., 2001; Tuettenberg et al., 2006), less is known about the potential use of tolerogenic DC in the clinic. It was shown that injection of tolerogenic DC into healthy volunteers is feasible and safe (Dhodapkar et al., 2001). Also initial approaches in the treatment of patients with type 1 diabetes have been successfully performed (Giannoukakis et al., 2011) showing that this method will also be attractive for immunotherapeutic strategies in autoimmune diseases, allergy and transplant rejection. Thus, DC vaccination research has to answer crucial questions to improve the efficacy of vaccination strategies. However, just like for immunogenic DC in anti-cancer trials, several quality criteria have to be defined for tolerogenic DC. Characteristics of tolerogenic DC like their migratory behavior or their functional stability after application *in vivo* need to be addressed in detail.

Translation of DC immunology into clinic implies generation of large amounts of donor-specific DC under good manufacturing practice (GMP)-conditions. Peripheral blood contains two major DC subsets, plasmacytoid and conventional DC, both with low frequencies (1–2% of PBMC) (Macdonald et al., 2002). On account of these low frequencies only a few vaccination studies used directly isolated DC subsets from leukapheresis products (Hsu et al., 1996; Tel et al., 2013). The fact that tumor patients show reduced DC frequencies in peripheral blood further exacerbates isolation of DC subpopulations (Savary et al., 1998). Therefore, initial approaches to expand patients peripheral DC by systemically administering, i.e., Flt3 ligand [a growth factor for myeloid and lymphoid DC progenitor cells (Lyman and Jacobsen, 1998)] have been performed (Fong et al., 2001). As an alternative, CD34^+^ hematopoietic stem cells isolated from blood or bone-marrow can be used for DC generation (Caux et al., 1997; Ueno et al., 2010). As CD34^+^ cells are represented only in low frequencies in peripheral blood, these cells are commonly mobilized into the blood by administration of G-CSF. Since this protocol is based on proliferating stem cells the resulting DC are rather heterogeneous and additional steps to improve purity are required.

As shown recently, monocytes serve as a pool of DC precursors *in vivo* that are recruited under inflammatory conditions like a bacterial infection (Cheong et al., 2010). Therefore, monocytes constitute an ideal cell population with a high abundance in peripheral blood that give rise to homogenous DC populations (Romani et al., 1994; Sallusto and Lanzavecchia, 1994). Several protocols for isolation of monocytes from PBMC exist such as positive selection via CD14 (Thurner et al., 1999) or counterflow centrifugation elutriation (Figdor et al., 1982). A very simple technique is the isolation of monocytes by their adherence to plastic surfaces, exemplarily described in this section. Usually, whole blood or buffy coats comprise sufficient PBMC numbers for monocyte isolation in daily routine experiments. However, repetitive treatments of patients with DC require large DC numbers. In this case PBMC isolation from a leukapheresis is a feasibly option (Jonuleit et al., 2001).

Many different protocols for generation of DC have been used in clinical studies, mostly based on the common protocol using GM-CSF and IL-4 for DC generation from monocytes (Romani et al., 1996). But also culture of monocytes in presence of GM-CSF and IL-15 leads to differentiation of DC resembling Langerhans cells (Anguille et al., 2009) that are able to induce tumor-specific T cell responses (Dubsky et al., 2007). However, both DC populations were comparably efficient in induction of anti-tumor T cell responses *in vivo* (Mohamadzadeh et al., 2001; Romano et al., 2011; Schuler, 2011).

**Figure 7:**
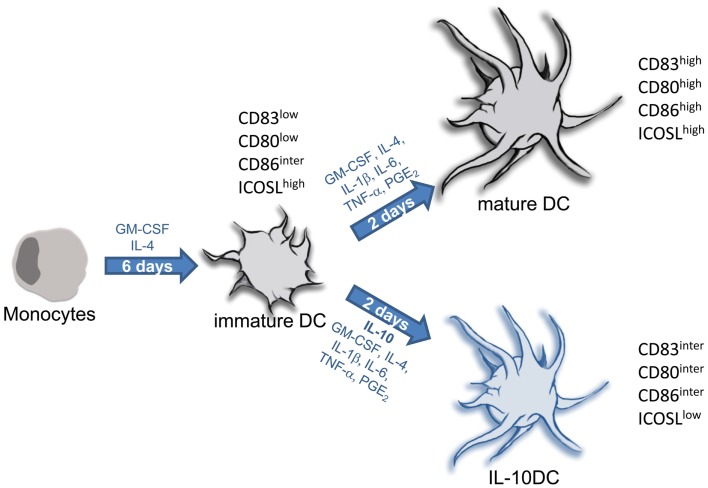
**Protocols for generation of tolerogenic and immunogenic dendritic cells from monocytic precursors**. In the presence of IL-4 and GM-CSF isolated monocytes differentiate into immature DC within 5–7 days. Terminal differentiation into fully mature DC is induced upon stimulation with inflammatory factors (IL-1, IL-6, and TNF) together with PGE_2_. This is a common protocol for generation of immunogenic DC used in numerous clinical trials for DC-based vaccination strategies in context of cancer. Maturation of DC carried out in presence of IL-10 provokes differentiation into tolerogenic IL-10DC.

In the following we will describe two protocols for the generation of monocyte-derived DC, tolerogenic versus immunogenic. The protocol for terminal differentiated mature DC has been successfully used in many studies by several investigators as an immunotherapeutic drug to treat cancer (Figure 7) (Tuettenberg et al., 2006; Correll et al., 2010).

### Isolation of peripheral blood mononuclear cells

Buffy coats or blood samples should be stored always at room temperature (RT) to prevent agglutination of lymphocytes and granulocytes. For isolation of PBMC by performing density gradient centrifugation follow these steps:

1. Prepare 10–15 ml of separation medium in a 50-ml tube. Take care to obey manufactures instruction for storage of the separation medium as temperature affects density.
2. Carefully layer 25 ml of blood/buffy coat onto the separation medium.
3. Centrifuge at 300 *g* for 30 min. at RT without brake.
4. Four phases should be visible. Top to bottom: plasma phase, interphase with PBMC, separation media, erythrocytes/granulocytes.
5. Convey plasma (if needed) and PBMC into separate new 50 ml tubes. At this moment PBMC might be contaminated with thrombocytes. Several washing steps are required to increase purity of PBMC.
6. For washing add 1× PBS (without Ca^2+^/Mg^2+^) + 0.5 mM EDTA ad 50 ml to PBMC.
7. Centrifuge at 400 *g* for 7 min and 4°C.
8. Repeat washing until supernatant is clear and then determine the number of isolated PBMC.

Isolated PBMC can directly be used for generation of DC. If PBMC are suspended in culture media and kept at 4°C, they can be stored overnight in X-VIVO-15 or RPMI1640 + 2% heat-inactivated plasma until use. PBMC can be stored for a longer time period when suspended in human serum albumin + 10% DMSO and frozen in liquid nitrogen.

### Collection and inactivation of plasma

Dependent on the culture medium, generation of DC from monocytes requires supplementation of culture media with autologous plasma. Plasma can be collected directly after density gradient centrifugation during PBMC isolation (see step 5 in Isolation of Peripheral Blood Mononuclear Cells). For inactivation of plasma proteins (e.g., complement factors), plasma is incubated in a water bath at 56°C for 30 min. The hereby degraded proteins are removed by centrifuging at 1500 *g* for 15 min. Note, first heat-inactivation, then centrifugation. Plasma can be stored at +4°C.

### Generation of immature dendritic cells from monocytes

*In vitro* generation of human DC from monocytes requires caution in terms of choosing the appropriate culture media. Several media meet the guidelines of GMP and can be used for DC generation. Successful studies have been performed using CellGro media (Cellgenix) or X-VIVO media (Lonza) (Schuler-Thurner et al., 2000; Royer et al., 2006; Schadendorf et al., 2006; Tuettenberg et al., 2006). Importantly, depending on local regulations for GMP media, there are major differences in the quality of generated DC. For example, X-VIVO-15 derived from Belgium is suited for generation of fully matured DC, whereas X-VIVO-15 produced in USA contains less serum proteins and generates more immature DC. Therefore, the particular objective dictates the particular media that should be used for DC generation. Likewise, RPMI1640 supplemented with autologous plasma can be employed (Thurner et al., 1999). The use of FCS with xenogeneic proteins for supplementation should be avoided because residual FCS-proteins can potentially induce FCS-IgE responses in patients (Jonuleit et al., 2000a, 2001).

We suggest using the following protocol for generation of substantial yields of immature DC (Figure 7) in which we use X-VIVO-15 (Lonza, Belgium) as culture media.

− Preheat all media used through the protocol to 37°C.
− Prepare RPMI1640 (Gibco) with 1.5% autologous, heat-inactivated plasma.
− Prepare X-VIVO-15 with 1% autologous, heat-inactivated plasma.

1. Transfer 10–15 × 10^6^ PBMC per well to a 6-well cell culture plate (polystyrene, wells treated for cell culture, e.g., Costar) in 2 ml of preheated RPMI1640 supplemented with 1% autologous plasma.
2. Incubate culture plates for 30–60 min at 37°C in an incubator. Place plates next to each other for optimal temperature adjustment. Monocytes start to produce an extracellular matrix that allows adherence to plastic surfaces.
3. Control grade of adherence under the microscope by slightly shaking the culture plate.
4. Carefully wash wells with preheated PBS and rinse off contaminating lymphocytes using a 10-ml pipette.
5. Adhered monocytes are cultured in 3 ml of preheated culture media, 400 IU/ml GM-CSF (growth factor for myeloid cells) and 1000 IU/ml IL-4 (prevents dominant differentiation of monocytes into macrophages). Cytokines in GMP-quality are available, i.e., from Cellgenix, Freiburg, Germany.
6. At day two and day four remove 1 ml of culture media from each well. Replace with 1 ml of preheated culture media supplemented with 800 IU/ml GM-CSF and 1000 IU/ml IL-4.
7. At day 6 immature DC can be harvested. Caution: immature DC are not terminally differentiated. Withdrawal of IL-4 supplementation leads to differentiation into macrophages.

### Terminal differentiation of immature dendritic cells

The maturation process of DC is induced by imitating an inflammatory situation in the skin. This is simply performed by addition of proinflammatory cytokines usually produced during inflammation: IL-1β, IL-6, and TNF-α. In addition, prostaglandin-E2 (PGE_2_) further accelerates DC maturation (Jonuleit et al., 1997; Steinbrink et al., 2000). Immature DC that are stimulated following this protocol do not produce IL-12p70, a cytokine involved in T cell polarization and NK cell activation that was thought to be crucial for proper anti-tumor T cell responses. However, clinical trials applying this protocol for DC maturation showed potent induction of cytotoxic tumor-reactive CD8^+^ T cells and Th1 polarization in patients. Modified protocols for DC maturation employing TLR ligands or type I interferons enable IL-12 production in DC that likewise initiate anti-tumor T cell responses in patients (Schuler, 2011; Wieckowski et al., 2011; Hansen et al., 2013).

To terminally differentiate immature DC:

1. 10^6^ immature DC are cultured in 3 ml preheated X-VIVO-15 supplemented with 1% plasma, 400 IU/ml GM-CSF, 1000 IU/ml IL-4, 1700 IU/ml IL-1β, 1000 IU/ml IL-6, 500 IU/ml TNF-α (i.e., Cellgenix), and 1 μg/ml PGE_2_ (Cayman Chemical Comp, USA) in a fresh 6-well culture plate.
2. After 48 h DC are terminally differentiated and can be harvested. Mature DC are non-adherent, therefore contamination with adherent cells should be avoided.

Clinical use of DC requires the determination of quality control criteria (Romani et al., 1996; Vries et al., 2002). Fully mature DC exhibit high and homogeneous expression of CD83, CD80, CD86, MHC class-I and class-II analyzed by flow cytometry (Figure 3A). Also functional testing is required, for this, alloreactive T cells are stimulated with different ratios of mature DC (Figure 2C). Half maximal T cell proliferation provoked by fully matured DC is achieved at DC:T cell ratios of approximately 1:200.

Phenotypic stability: as mature DC are terminally differentiated this can easily be tested by performing a cytokine washout. For this, mature DC are harvested, washed and plated in a fresh 6-well plate in preheated culture medium in absence of additional cytokines. Terminally differentiated mature DC still conform to quality issues after 24–48 h of culture in absence of cytokines (Figdor et al., 2004).

### Generation of tolerogenic IL-10-modulated dendritic cells

As noted, tolerogenic properties of immature DC are strengthened by modulation with IL-10 (Steinbrink et al., 1997). IL-10 is a very sensitive cytokine; its biological activity is preserved best by storage at −80°C and is often lost within 24 h after thawing. For generation of IL-10DC the following protocol is suggested.

1. Incubate 15 × 10^6^ PBMC per well of a 6-well-plate in 2 ml RPMI1640 + 1% plasma for 30 min in an incubator.
2. Rinse off non-adherent cells by washing wells with 1× PBS.
3. Subsequently, remaining adherent cells are cultured in 2 ml X-VIVO-15 + 1% plasma overnight.
4. On day 1, replace media with 3 ml fresh X-VIVO-15 supplemented with 400 IU/ml GM-CSF, 150 IU/ml IL-4, and 1% plasma.
5. On day 3, replace 1 ml media with 1ml fresh X-VIVO-15 supplemented with 800 IU/ml GM-CSF, 150 IU/ml IL-4, and 1% plasma.
6. On day 6, immature DC can be harvested.
7. 10^6^ immature DC are cultured in 3 ml X-VIVO-15 supplemented with 1% plasma, 400 IU/ml GM-CSF, and 150 IU/ml IL-4. For differentiation into IL-10DC 5 ng/ml IL-1β, 5 ng/ml TNF-α (e.g., Miltenyi Biotec, Germany), 50 IU/ml IL-6, 1 μg/ml PGE_2_, and 20 ng/ml IL-10 (e.g., Schering-Plough Corporation) are added.
8. Stable tolerogenic IL-10DC can be harvested 48 h later.

In conclusion, a successful immunotherapeutic strategy will include a combination of DC vaccination with additional therapies targeting other immune cell populations as well as the tumor or tumor micromilieu itself. The combination of DC-modulating agents with additional therapies such as antibody treatment to inactivate or deplete Treg could possibly increase the potential of DC-based immunotherapy. Some clinical studies using Treg-depletion in tumor patients followed by DC vaccination showed that elimination of Treg enhances the magnitude of tumor-specific T cell responses. Finally, these issues need to be addressed in comparative clinical studies to determine optimal vaccine characteristics. DC vaccination can then be put to the ultimate test in randomized clinical trials. The same is true for the use of tolerogenic DC in terms of treating an over activated immune system in context of autoimmunity. Also here, application of tolerogenic DC in combination with modulation of proinflammatory mediators could possibly synergize in action.

Importantly, the described protocol for generation of immunogenic DC is commonly used for initiation of anti-melanoma responses. It can be suspected that a different target tumor could require a different maturation protocol or even a different protocol for DC generation. Therefore, analyses addressing the impact of various protocols for DC generation and maturation on the resulting T effector cell populations are of importance and could improve DC-based therapeutic success.

## Conflict of Interest Statement

The authors declare that the research was conducted in the absence of any commercial or financial relationships that could be construed as a potential conflict of interest.
